# Log odds of positive lymph nodes as a novel prognostic predictor for gastric cancer: a systematic review and meta-analysis

**DOI:** 10.1186/s12885-023-10805-6

**Published:** 2023-06-08

**Authors:** Yiding Li, Guiling Wu, Jinqiang Liu, Yujie Zhang, Wanli Yang, Xiaoqian Wang, Lili Duan, Liaoran Niu, Junfeng Chen, Wei Zhou, Weili Han, Jing Wang, Helun Zhong, Gang Ji, Daiming Fan, Liu Hong

**Affiliations:** 1 Department of Digestive Surgery, Xijing Hospital, Air Force Medical University, 127 Changle West Road, Xi’an, Shaanxi Province 710032 China; 2 School of Aerospace Medicine, Fourth Military Medical University, Xi’an, 710032 China; 3 Department of Histology and Embryology, School of Basic Medicine, Xi’an Medical University, Xi’an, 710021 China; 4 Department of Immunology, Fourth Military Medical University, Xi’an, 710032 China; 5 Treatment Centre for Traumatic Injures, Academy of Orthopedics Guangdong Province, The Third Affiliated Hospital of Southern Medical University, Guangzhou, 510630 China

**Keywords:** Log odds of positive lymph nodes (LODDS), Gastric cancer, Prognostic predictor, Overall survival, Meta-analysis

## Abstract

**Background:**

We conducted a systematic review and meta-analysis to summarize the predictive and prognostic ability of the log odds of positive lymph nodes (LODDS) staging system and compare it with pathological N (pN) classification and the ratio-based lymph node system (rN) for the overall survival (OS) of gastric cancer (GC).

**Methods:**

Through a systematic review till March 7, 2022, we identified population-based studies that reported the prognostic effects of LODDS in patients with GC. We compare the predictive effectiveness of the LODDS staging system with that of the rN and pN classification systems for the OS of GC.

**Results:**

Twelve studies comprising 20,312 patients were included in this systematic review and meta-analysis. The results showed that LODDS1, LODDS2, LODDS3, and LODDS4 in GC patients were correlated with poor OS compared with LODDS0 (LODDS1 vs. LODDS0: HR = 1.62, 95% CI (1.42, 1.85); LODDS2 vs. LODDS0: HR = 2.47, 95% CI (2.02, 3.03); LODDS3 vs. LODDS0: HR = 3.15, 95% CI (2.50, 3.97); LODDS4 vs. LODDS0: HR = 4.55, 95% CI (3.29, 6.29)). Additionally, significant differences in survival were observed among patients with different LODDS classifications (all *P*-values were < 0.001) with the same rN and pN classifications. Meanwhile, for patients with different pN or rN classifications with the same LODDS classification, prognosis was highly similar.

**Conclusion:**

The findings show that LODDS is correlated with the prognosis of GC patients and is superior to the pN and rN classifications for prognostic assessment.

**Supplementary Information:**

The online version contains supplementary material available at 10.1186/s12885-023-10805-6.

## Introduction

Gastric cancer (GC), a common highly recurrent malignant tumor, is the fifth most prevalent tumor, and is the third most frequent primary cause of tumor-related death worldwide [1]. Multiple markers or methods predict the prognosis and therapeutic outcomes of patients with GC. Among these, nodal involvement has long been considered as one of the greatest maker for prognosis and metastasis [2, 3]. For this reason, the International Union Against Cancer (UICC) and the American Joint Committee on Cancer (AJCC) have adopted categories based on the number of metastatic lymph nodes (LNs) as the basis for the N stage of the tumor–node–metastasis (TNM) classification. The TNM staging system is the most widely used system for GC staging assessment. For optimal staging of GC, the latest edition recommends the examination of 16 or more LNs for nodal metastatic status determination [4]. Unfortunately, poor compliance was demonstrated with the recommendation: > 15 LNs were removed in only 29% of patients, and no nodes were removed at all in 9% [5–7]. Multiple studies have demonstrated that the accuracy of pathological N (pN) classification for patients’ prognostic assessment is affected by the number of examined LNs [6, 8]. Particularly, if the number of examined LNs is less than 16, N3b patients may be inappropriately classified as N3a because the cutoff for N3a/N3b is 15 metastatic LNs [9]. This phenomenon has been referred to as stage migration. To overcome the potential bias associated with the pN classification, other parameters have been proposed. Some authors have claimed that the ratio-based lymph node system (rN) can be an alternative for patients with fewer than 15 LNs examined, defined as the ratio of metastatic LNs to examined LNs [10–12]. Chen et al. showed that lymph node ratio (LNR) carries more information than the pN classification in patients with an inadequate node count [13]. However, the rN0 classification was congruent with the pN0 classification in prognostic assessments of node-negative patients with GC [12, 14]. For this reason, another novel solution, the log odds of positive lymph nodes (LODDS), was proposed recently [11]. LODDS, as a novel prognostic indicator, is defined as the log of the ratio between the number of positive nodes and the number of negative nodes and further discriminates patients with N0 GC [14, 15]. LODDS, first proposed in breast cancer in which it performed equally well as a prognostic indicator in node-positive and node-negative patients [16], was later generalized to several cancers, including GC [17–22]. The LODDS classification was superior in predicting the prognosis of GC patients with < 15 examined LNs and those with N0 status compared with the rN and pN classifications [11, 23–25]. However, the studies supporting this finding have several limitations, including a lack of large, multicenter surveillance studies and available inclusion criteria for patients and different predicative capabilities of LODDS in different studies.

We conducted a systematic review and meta-analysis to summarize the predictive and prognostic ability of the LODDS staging system and compare it with the rN and pN classification systems to address these limitations.

## Methods

### Data sources

We searched PubMed, Medline, Embase, Web of Science and the Cochrane Library for relevant studies from inception to March 7, 2022, which formed the basis for evidence used to conduct the meta-analysis. The following keywords were used: “log odds of positive lymph nodes”, “LODDS”, and “gastric cancer”. We used the following strategy: (((((((((gastric neoplasms[MeSH Terms]) OR (stomach neoplasms)) OR (stomach carcinoma)) OR (gastric tumor)) OR (gastric carcinoma)) OR (stomach cancer)) OR (stomach tumor)) OR (gastric neoplasms)) OR (GC)) AND ((log odds of positive lymph nodes) OR (LODDS)). We followed the Preferred Reporting Items for Systematic Reviews and Meta-Analyses (PRISMA) statement for reporting systematic reviews [26] and registered our review on Prospero (https://www.crd.york.ac.uk/prospero/). The PROSPERO registration number is CRD42021274996.

### Inclusion and exclusion criteria

We included studies that enrolled ≥ 100 patients with GC (diagnosed with the gold standard test) who were classified by the LODDS, rN, and pN classification systems of the AJCC and followed for at least five years. We included studies that reported at least one of the outcomes of interest or studies wherein the outcome could be calculated according to data extracted from the published data. We excluded conference abstracts, reviews, case reports, ongoing trials, open-label trials, comments, letters, meeting records, and studies that enrolled patients with a total sample size of less than 100 patients, as studies with very small samples are more prone to bias and contribute little information to pooled analyses. We excluded articles that shared a study population with another article and those that did not provide crucial information needed for detailed stratification.

### Study selection

Using a standardized form, two reviewers independently screened the titles and abstracts identified by the search, and the full text was obtained to check eligibility. When a disagreement arose and they were unable to reach a consensus, an adjudicator was consulted to resolve discrepancies.

### Data extraction

Using a standardized form, two reviewers worked independently to perform duplicate data abstraction in each eligible study. When a disagreement arose and they were unable to reach a consensus, an adjudicator helped to resolve disagreements. We collected information regarding study characteristics (including year of publication, single-center/multicenter study, clinical study design, country of patients, and patient year), patient characteristics (including patient number, tumor stage, patient age, population, neoadjuvant therapy, number of harvested LNs, and number of metastatic LNs), and all patient-important outcomes (overall survival (OS)).

### Outcomes and quality assessment

Prognostic values (OS) were used to compare the different LODDS groups. We compared the predictive and prognostic abilities of the LODDS staging system with those of the rN and pN classification systems.

Using the Newcastle–Ottawa Scale (NOS) [27], two investigators, independently and in duplicate, assessed the quality of the included articles, including study group selection, comparability of groups and outcome of interest. The full score was 9 points, and a score of 1–4 points indicated low-quality, while a score of 5–9 points indicated high-quality.

### Data analysis and statistical methods

The data were analyzed using Stata statistical software version 16.0 (Stata Corporation, College Station, TX) and SPSS version 22 (IBM Corporation).

To statistically assess the prognostic effects of LODDS, we extracted the hazard ratio (HR) and 95% confidence interval (CI) of 5-year OS from the included studies. If not directly provided in the original literature, the estimated HR and 95% CI were used to assess prognostic effects based on the method described by Tierney et al. [28], and an HR greater than 1 suggested a higher risk of disease progression or death in the patients. A random-effects model (REM) was used to pool the data. All statistical values were combined with 95% CIs and *P* values for two-sided testing at α = 0.05. To assess the existence of heterogeneity among studies, we tested OS for heterogeneity across cohorts using Cochran’s Q statistic and used *I*^*2*^ to measure the extent of heterogeneity [29]. For the *I*^*2*^ statistic, heterogeneity was defined as low (25%–50%), moderate (50%–75%) or high (> 75%) [30]. For the Q statistic, *P* ≤ 0.1 was considered to indicate significant heterogeneity. In addition, subgroup analyses were performed based on the differences in baseline characteristics and risk factors in the data retrieved. Then, we also conducted a sensitivity analysis in which each study was removed in turn to evaluate the undue influence of the study on the overall summary estimates including Duval and Tweedie’s trim-and-fill method [31], and Galbraith plots [32]. Publication bias was investigated with qualitative and quantitative methods, including funnel plots and Egger’s test [33]. The groups in each pN and rN stage were regrouped in accordance with LODDS, and the OS differences within groups and between groups were analyzed using chi-squared tests to compare the homogeneity of the three staging methods. *P* values for the pooled results were two-sided, and the significance level was 0.05.

## Results

### Study characteristics

**Fig. 1:**
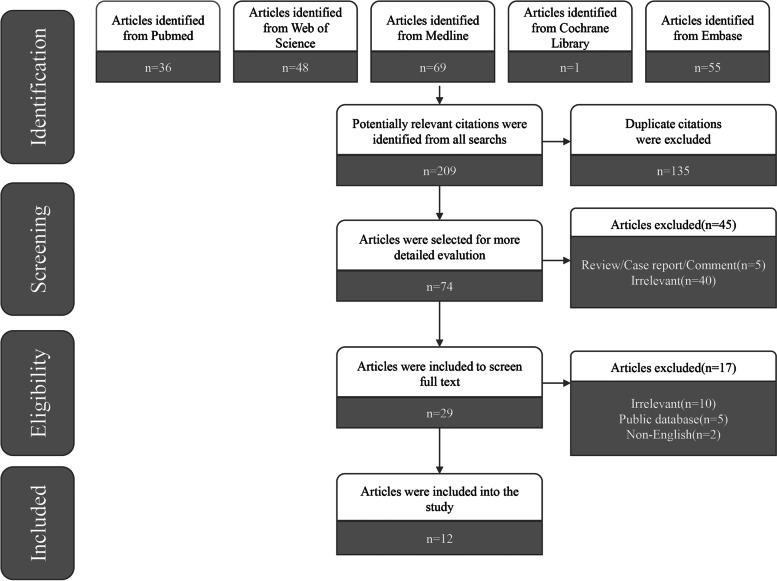
Flow diagram of study selection

Based on the search strategy mentioned above, the original search yielded 209 records. Finally, we included 12 unique studies [11, 12, 14, 15, 23–25, 34–38] published between 2010 and 2021 with 20,312 patients according to the eligibility criteria. The flowchart of the search and selection process is shown as a PRISMA flowchart in Fig. 1.

Tables 1 and 2 summarizes the main characteristics of the studies included in the meta-analysis. Because of the different hierarchical levels of LODDS in the included studies, we re-stratified the groups in the meta-analysis. Eleven studies were retrospective, and one study used a prospectively maintained database. Among these studies, eight studies were from China [11, 12, 14, 23, 24, 35, 37, 38], and one study each was from Hungary [25], Korea [34], America [15], and Italy [36]. Among the included studies, only five studies [11, 24, 25, 35, 36] included patients without neoadjuvant therapy, three studies [15, 23, 37] did not limit the number of patients who received or did not receive neoadjuvant therapy, and the remaining studies [12, 14, 34, 38] did not report the relevant information. Four studies [11, 12, 14, 38] compared the predictive and prognostic abilities of the LODDS staging system with those of the rN or pN classification systems.

**Table 1:** Characteristics of included studies for the meta-analyses

Reference	Year	Single-center/multicenter	Clinical study design	Country	Patient year	Patient		Tumor stage	Age(years)	Population	Neoadjuvant therapy
						Number	Male/female				
Gu P [11]	2021	Multicenter	retrospective	China	2001–2011	7620	5378/2242	I-III	Median (range): 58.3 (15–89)	undergone curative gastric cancer resection with lymphadenectomy (R0 resection)	No
Pan S [23]	2019	single-center	retrospective	China	1987–2012	1730	1250/480	I-III	Median (IQR): 58 (50–66)	underwent radical gastrectomy	Yes
Cao H [24]	2019	single-center	retrospective	China	2010–2012	877	605/272	I-III	-	gastric adenocarcinoma R0 resection with D2 LN dissection	No
Toth D [25]	2017	Multicenter	retrospective	Hungary	2005–2010	164	99/65	I-IV	Median (range): 66 (35–90)	gastric adenocarcinoma R0 resection	No
Lee JW [34]	2016	single-center	retrospective	Korean	1990–2012	3929	2623/1306	NC	Median (range): 59 (18–91)	underwent curative (R0) resection and extended lymphadenectomy	UKN
Jian-Hui C [35]	2016	single-center	retrospective	China	1994–2008	935	636/299	NC	mean (range): 57.5 (24–87)	gastric adenocarcinoma R0 resection with D2 LN dissection	No
Spolverato G [15]	2015	Multicenter	retrospective	America	2000–2012	804	468/336	NC	Median (IQR): 66.1 (56.7–74.3)	underwent surgical resection of gastric adenocarcinoma	Yes
Aurello P [36]	2014	single-center	retrospective	Italy	1997–2012	177	107/70	NC	mean (range): 65.9 (32–88)	gastric adenocarcinoma who underwent curative gastrectomy (R0)	No
Xu J [12]	2013	single-center	retrospective	China	2005–2008	427	281/146	NC	-	underwent standard D2 radical gastrectomy	UKN
Liu H [37]	2013	single-center	retrospective	China	2001–2006	372	240/132	NC	mean (range): 58.9 (20 − 79)	gastric adenocarcinoma R0 resection with D2 LN dissection and number of lymph nodes retrieved were no less than 15	Yes
Qiu MZ [38]	2012	single-center	retrospective	China	1996–2006	730	522/208	I-III	mean (range): 60 (24 − 83)	receiving D2 resection carried out by experienced surgeons in our hospital following the Japanese Gastric Cancer Association (JGCA) guidelines	UKN
Sun Z [14]	2010	single-center	prospectively	China	1980–2008	2547	-	-	-	underwent D2 or D3 lymphadenectomy and achieved radical (R0) resection for histologically proven gastric carcinoma	UKN

*Abbreviations*: *IQR* interquartile range, *UKN* Unknown

- not available

**Table 2:** Clinicopathologic characteristics of included studies for the meta-analyses

Reference	Year	The number of harvested LNs	The number of metastatic LNs	UICC/AJCC TNM	Follow − up(mouth)	Follow-up	Cutoff	Groups in the study					NOS score
								LODDS0	LODDS1	LODDS2	LODDS3	LODDS4	
Gu P [11]	2021	Median (range): 58.3 (15–89)	Median (range): 2.0 (0–84)	the 8th edition	Median (range): 87 (2 − 186)	5-year OS	LODDS 0 (LODDS ≤ -1.5) LODDS 1 (-1.5 < LODDS ≤ -1.0) LODDS 2 (-1.0 < LODDS ≤ -0.5) LODDS 3 (-0.5 < LODDS ≤ 0) LODDS 4 (LODDS > 0)	1992	1567	1474	1409	1178	8
Pan S [23]	2019	Median (IQR): 21 (13–32)	Median (IQR): 1 (0–4)	the 8th edition	Median (IQR): 36.0 (1 − 426)	5-year OS	LODDS 0 (LODDS ≤ -1.5) LODDS 1 (-1.5 < LODDS ≤ -1.0) LODDS 2 (-1.0 < LODDS ≤ -0.5) LODDS 3 (-0.5 < LODDS ≤ 0) LODDS 4 (LODDS > 0)	506	443	324	303	154	8
Cao H [24]	2019	-	-	the 8th edition	-	5-year OS	LODDS 0 (LODDS ≤ -0.5) LODDS 1 (-0.5 < LODDS ≤ 0) LODDS 2 (0 < LODDS ≤ 0.5) LODDS 3 (LODDS > 0.5)	261	-	160	312	144	7
Toth D [25]	2017	mean (range): 10.48 (1–38)	mean (range): 3.22 (0–23)	the 6th, the 7th, and the 8th edition	Median (range): 23.0 (3–136)	5-year OS	LODDS 0 (LODDS < –1.125) LODDS 1 (–1.125 ≤ LODDS < –0.25) LODDS 2 (–0.25 ≤ LODDS < 0.75) LODDS 3 (LODDS ≥ 0.75)	29	-	75	43	17	8
Lee JW [34]	2016	mean ± SD: 41.2 ± 16.5	mean ± SD: 3.22 ± 6.8	the 7th edition	Median: 69.7	5-year OS	LODDS 0 (LODDS ≤ -4) LODDS 1 (-4 < LODDS ≤ -2.5) LODDS 2 (-2.5 < LODDS ≤ -2) LODDS 3 (-2.5 < LODDS ≤ -2) LODDS 4 (LODDS > -0.5)	1850	996	226	609	248	8
Jian-Hui C [35]	2016	mean (range): 24.9 (0–140)	mean (range): 4.61 (0 − 124)	the 7th edition	Median (range): 53.3 (1.3–148.4)	5-year OS	LODDS 0 (LODDS ≤ -1.5) LODDS 1 (-1.5 < LODDS ≤ -1.0) LODDS 2 (-1.0 < LODDS ≤ -0) LODDS 3 (LODDS > 0)	277	170	385		103	6
Spolverato G [15]	2015	Median (IQR): 17 (11–25)	-	the 7th edition	Median: 21	5-year OS	LODDS 0 (LODDS ≤ -1.5) LODDS 1 (-1.5 < LODDS ≤ -1.0) LODDS 2 (-1.0 < LODDS ≤ -0.5) LODDS 3 (-0.5 < LODDS ≤ 0) LODDS 4 (LODDS > 0)	459	61	63	87	132	8
Aurello P [36]	2014	mean (range): 26.4 (3 − 73)	mean (range): 6.9 (0–59)	the 7th edition	Median: 77	5-year OS	LODDS 0 (LODDS < -1.0) LODDS 1 (-1.0 ≤ LODDS < -0.5) LODDS 2 (-0.5 ≤ LODDS < 0) LODDS 3 (0 ≤ LODDS < 0.5) LODDS 4 (LODDS ≥ 0.5)	88	27	31	19	12	8
Xu J [12]	2013	-	-	the 7th edition	Median (range): 55 (39–81)	5-year OS	LODDS 0 (LODDS < -1.0) LODDS 1 (-1.0 ≤ LODDS < -0.5) LODDS 2 (-0.5 ≤ LODDS < 0) LODDS 3 (0 ≤ LODDS < 0.5) LODDS 4 (LODDS ≥ 0.5)	129	87	85	76	50	8
Liu H [37]	2013	Median (range): 21 (15 − 66)	Median (range): 3 (0–21)	the 7th edition	Median (range): 52 (3–89)	5-year OS	LODDS 0 (LODDS < -1.5) LODDS 1 (-1.5 ≤ LODDS < -1.0) LODDS 2 (-1.0 ≤ LODDS < -0.5) LODDS 3 (LODDS ≥ -0.5)	100	64	71	137		8
Qiu MZ [38]	2012	Median (range): 16 (0 − 72)	Median (range): 4 (0–70)	the 7th edition	Median (range): 48 (3 − 175)	5-year OS	LODDS 0 (LODDS < -0.5) LODDS 1 (-0.5 ≤ LODDS < 0) LODDS 2 (0 ≤ LODDS < 0.5) LODDS 3 (LODDS ≥ 0.5)	305	174		142	109	8
Sun Z [14]	2010	-	-	the 6th edition	Median (range): 41 (3 − 332)	5-year OS	LODDS 0 (LODDS ≤ -1.5) LODDS 1 (-1.5 < LODDS ≤ -1.0) LODDS 2 (-1.0 < LODDS ≤ -0.5) LODDS 3 (-0.5 < LODDS ≤ 0) LODDS 4 (LODDS > 0)	544	594	533	528	348	8

*Abbreviations*: *OS* overall survival, *LODDS* log odds of positive lymph nodes, *IQR* interquartile range, *SD* Standard Deviation

- not available

### Study analysis

#### LODDS and OS in GC

We analyzed OS in different LODDS categories according to the data from the included articles. The results of the pooled analysis are summarized in Table 3.

**Table 3:** Results of subgroup analyses on prognostic effects of GC patients

Comparisons (vs LODDS0)	OS				
	No. of studies	HR (95% CI)	Heterogeneity		Egger’s Test *p* Value
			I^2^ (%)	*P*	
**TOTAL studies**					
LODDS1	10	1.62 (1.42, 1.85)	63.5%	0.003	0.608
LODDS2	11	2.47 (2.02, 3.03)	86.2%	< 0.001	0.799
LODDS3	11	3.15 (2.50, 3.97)	92.1%	< 0.001	0.943
LODDS4	11	4.55 (3.29, 6.29)	96.6%	< 0.001	0.216
**Year**^**c**^					
≥ Median					
LODDS1	5	1.50 (1.23, 1.82)	79.4%	0.001	
LODDS2	6	2.41 (1.88, 3.09)	83.3%	< 0.001	
LODDS3	6	2.95 (2.04, 4.27)	95.7%	< 0.001	
LODDS4	6	5.82 (4.30, 7.87)	90.8%	< 0.001	
< Median					
LODDS1	5	1.78 (1.56, 2.04)	0.0%	0.714	
LODDS2	5	2.58 (1.76, 3.80)	89.3%	< 0.001	
LODDS3	5	3.38 (2.80, 4.09)	53.9%	0.07	
LODDS4	5	3.32 (2.11, 5.23)	95.9%	< 0.001	
**Country**					
East Asia					
LODDS1	8	1.65 (1.49, 1.84)	39.9%	0.113	
LODDS2	8	2.56 (2.15, 3.05)	77.5%	< 0.001	
LODDS3	8	3.33 (2.86, 3.87)	77.8%	< 0.001	
LODDS4	8	4.89 (3.90, 6.14)	90.6%	< 0.001	
non-East Asia					
LODDS1	2	1.57 (0.69, 3.57)	86.7%	0.006	
LODDS2	3	2.21 (1.04, 4.71)	88.7%	< 0.001	
LODDS3	3	2.79 (1.21, 6.44)	90.2%	< 0.001	
LODDS4	3	3.60 (1.55, 8.32)	90.1%	< 0.001	
**Patient no.**^**d**^					
≥ Median					
LODDS1	5	1.60 (1.42, 1.81)	56.0%	0.059	
LODDS2	6	2.47 (2.01, 3.04)	83.2%	< 0.001	
LODDS3	6	3.23 (2.71, 3.85)	83.7%	< 0.001	
LODDS4	6	4.05 (2.63, 6.24)	98.0%	< 0.001	
< Median					
LODDS1	5	1.76 (1.24, 2.49)	73.7%	0.004	
LODDS2	5	2.50 (1.55, 4.03)	87.0%	< 0.001	
LODDS3	5	3.17 (1.77, 5.68)	92.0%	< 0.001	
LODDS4	5	5.43 (3.04, 9.69)	91.0%	< 0.001	
**Neoadjuvant therapy**					
No					
LODDS1	3	1.85 (1.64, 2.07)	0.0%	0.381	
LODDS2	5	3.04 (2.58, 3.58)	29.2%	0.227	
LODDS3	4	4.03 (3.66, 4.43)	0.0%	0.975	
LODDS4	5	6.36 (4.50, 8.99)	81.3%	< 0.001	
All					
LODDS1	3	1.38 (1.05, 1.82)	67.1%	0.048	
LODDS2	3	2.00 (1.39, 2.88)	87.5%	< 0.001	
LODDS3	3	2.47 (1.42, 4.30)	95.6%	< 0.001	
LODDS4	2	2.41 (1.34, 4.36)	97.5%	< 0.001	
UKN					
LODDS1	4	1.59 (1.35, 1.89)	49.0%	0.118	
LODDS2	3	2.26 (1.54, 3.31)	83.0%	0.003	
LODDS3	4	3.07 (2.53, 3.71)	66.2%	< 0.001	
LODDS4	4	4.29 (3.17, 5.82)	86.4%	< 0.001	

*Abbreviations*: *GC* Gastric cancer, *HR* hazard ratio, *OS* overall survival, *DFS* disease-free survival, *LODDS* log odds of positive lymph nodes, *UKN* Unknown

^a^*P*-value for estimates of HR

^b^*P*-value for heterogeneity

^c^The median year of LODDS1, LODDS2, LODDS3, and LODDS4 was 2015, 2016, 2015, and 2016, respectively

^d^The median patient number of LODDS1, LODDS2, LODDS3, and LODDS4 was 172, 160, 142, and 132, respectively

**Fig. 2:**
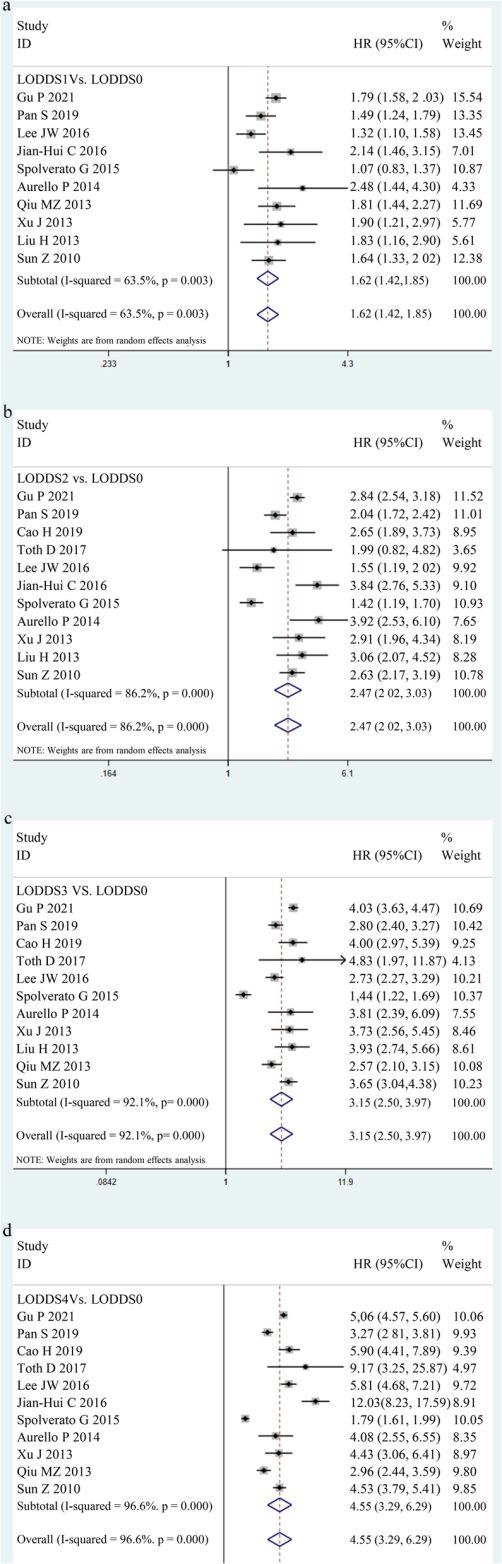
Estimated HR summary for OS. **a** LODDS1 vs. LODDS0, **b** LODDS2 vs. LODDS0, **c** LODDS3 vs. LODDS0, **d** LODDS4 vs. LODDS0. HR > 1 indicates more disease progression or deaths in the patients. Data were pooled using a random-effects model (REM). All statistical values were combined with 95% CIs and two-sided *P*-values, the threshold of which was set to 0.05. Abbreviations: HR, hazard ratio; OS, overall survival; LODDS, log odds of positive lymph nodes

When pooling the HR for OS, LODDS1, LODDS2, LODDS3, and LODDS4 in GC patients were correlated with poor OS compared with LODDS0 (Fig. 2). Patients with LODDS1 had inferior OS compared with those with LODDS0 (HR = 1.62, 95% CI (1.42, 1.85)), and the heterogeneity was significant (*I*^*2*^ statistic = 63.5%, P _*heterogeneity*_ = 0.003). The pooled results indicated that LODDS2 GC patients had a worse OS (HR = 2.47, 95% CI (2.02, 3.03)) than LODDS0 GC patients. There was significant heterogeneity (*I*^*2*^ statistic = 86.2%, *P *_*heterogeneity*_ < 0.001). Compared with LODDS0 GC patients, LODDS3 GC patients had a worse OS (HR = 3.15, 95% CI (2.50, 3.97)), and the heterogeneity was significant (*I*^*2*^ statistic = 92.1%, *P *_*heterogeneity*_ < 0.001). The results of the pooled analysis using the REM showed that LODDS4 GC patients were also associated with poorer OS (HR = 4.55, 95% CI (3.29, 6.29)) than LODDS0 GC patients, and between-study heterogeneity was significant (*I*^*2*^ statistic = 96.6%, P _*heterogeneity*_ < 0.001). Overall, as the LODDS grade increases, the prognosis of patients with GC becomes increasingly worse.

### Subgroup analysis

To analyze the potential sources of between-study heterogeneity, we performed subgroup analysis according to differences in the variables, including the publication year, country, patient number and whether patients received neoadjuvant therapy. The results are summarized in Table 3. Consistent with the above results, LODDS1, LODDS2, LODDS3, and LODDS4 GC patients had a worse OS than LODDS0 GC patients in all subsets. After subgroup analysis, it was found that the heterogeneity was mainly from whether patients received neoadjuvant therapy. In patients without neoadjuvant therapy, the result of the pooled analysis using the REM showed that LODDS1, LODDS2, LODDS3, and LODDS4 GC patients were also associated with poor OS (LODDS1 vs. LODDS0: HR = 1.85, 95% CI (1.64, 2.07); LODDS2 vs. LODDS0: HR = 3.04, 95% CI (2.58, 3.58); LODDS3 vs. LODDS0: HR = 4.03, 95% CI (3.66, 4.43); LODDS4 vs. LODDS0: HR = 6.36, 95% CI (4.50, 8.99)) compared with LODDS0 GC patients. The heterogeneity of OS decreased to a nonsignificant level except for patients in the LODDS4 vs. LODDS0 group (LODDS1 vs. LODDS0: *I*^*2*^ statistic = 0.0%, P _*heterogeneity*_ = 0.381; LODDS2 vs. LODDS0: *I*^*2*^ statistic = 29.2%, P _*heterogeneity*_ 0.227; LODDS3 vs. LODDS0: *I*^*2*^ statistic = 0.0%, P _*heterogeneity*_ = 0.975; LODDS4 vs. LODDS0: *I*^*2*^ statistic = 81.3%, P _*heterogeneity*_ < 0.001).

**Fig. 3:**
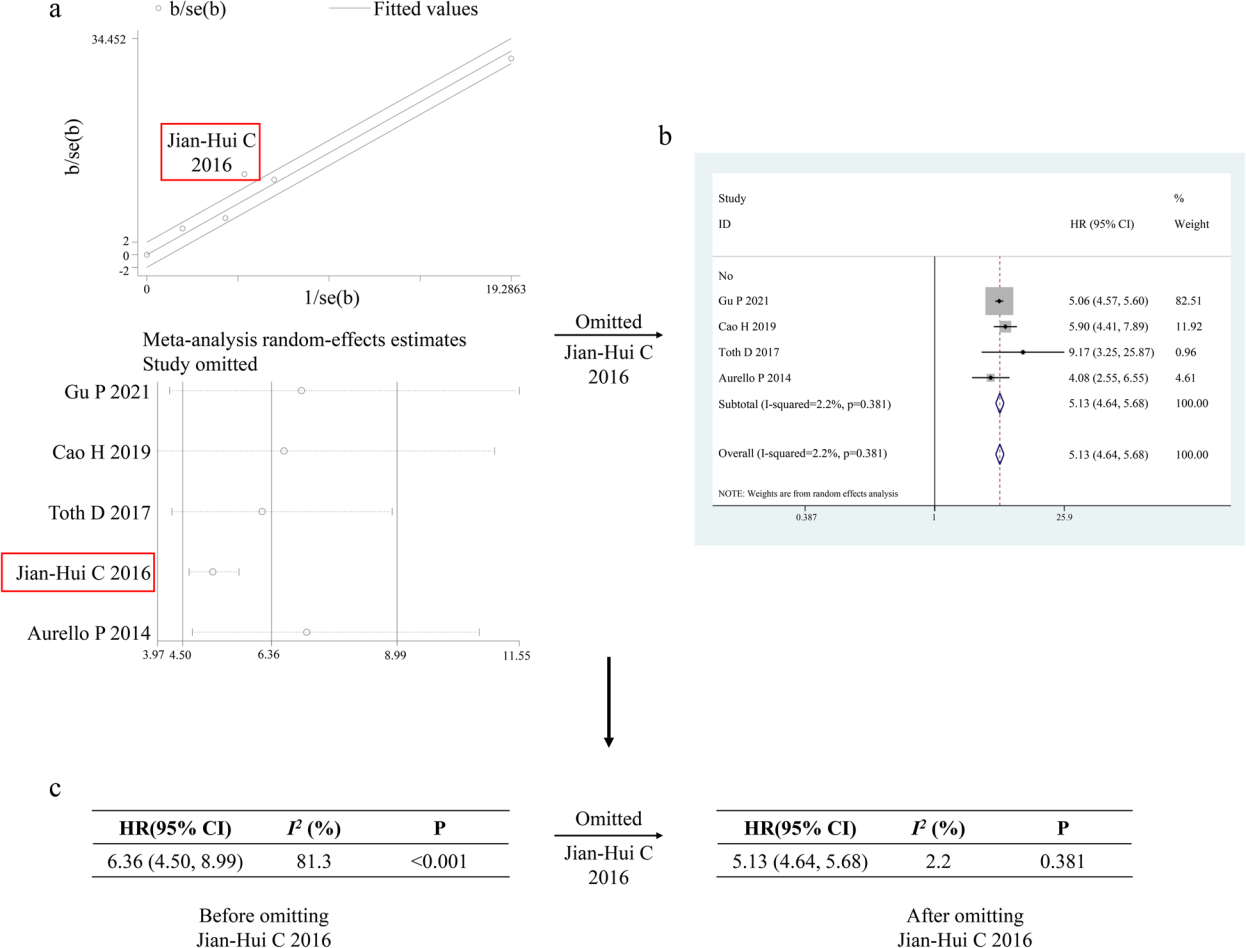
Process of exploring the potential sources of heterogeneity on OS. **a** galbraith plot for OS. **b** Forest plot for OS after Jian-Hui C et al. (2016) is omitted. **c** change of heterogeneity before and after Ogawa S et al. (2016) is omitted**.** Weights are from random-effects analysis. *P* value for heterogeneity. Abbreviations: HR, hazard ratio; OS, disease-free survival; SE, standard error

To explore the potential sources of heterogeneity in the LODDS4 vs. LODDS0 group, we also used Galbraith plots and Duval and Tweedie’s trim-and-fill method to further explore the source of heterogeneity in OS, and the results showed that the training set of the study by Jian-Hui C et al. [35] might have mainly contributed substantial heterogeneity to OS (Fig. 3a). After omitting this study, the pooled HR was not obviously affected (HR = 5.13, 95% CI (4.46, 5.68); Fig. 3b), but the heterogeneity for OS dropped to a nonsignificant level (from *I*^*2*^ statistic = 81.3%, P _*heterogeneity*_ < 0.001 to *I*^*2*^ statistic = 2.2%, P _*heterogeneity*_ = 0.381; Fig. 3c).

### Publication bias

Publication bias was assessed by funnel plots and Egger’s test. Formal evaluation using Egger’s test failed to identify significant publication bias in the analyses of LODDS1 vs. LODDS0 (*p* = 0.608), LODDS2 vs. LODDS0 (*p* = 0.799), LODDS3 vs. LODDS0 (*p* = 0.943), and LODDS4 vs. LODDS0 (*p* = 0.216) for OS. The results with *P* values for Egger’s test are listed in Table 3. In addition, we used funnel plots to detect publication bias, as shown in Figure S1. All of the funnel plots of the included articles showed a symmetrical distribution. Thus, no significant publication bias was found in the meta-analyses of OS.

### Comparison of the homogeneity of prognostic assessments

Four studies [11, 12, 14, 38] compared the predictive and prognostic abilities of the LODDS staging system with those of the rN or pN classification systems. OS rates were compared among different pN and rN classifications when stratified by the LODDS classification and among different LODDS classifications when stratified by the pN or rN classification. The data from all these studies were therefore pooled, as shown in Table 4. Thus, for patients in each of the pN classifications, significant differences in survival were consistently observed among patients with different LODDS classifications. And, for patients in each of the rN classifications, significant differences in survival were consistently observed among patients with different LODDS classifications. Meanwhile, for patients in each LODDS classification, prognosis was highly homologous for those with different pN or rN classifications. These results indicated that the LODDS classification might be superior to the pN and rN classifications for prognostic assessment.

**Table 4:** Overall survival rates according to different pN and rN classifications stratified by the LODDS staging system

	LODDS0		LODDS1		LODDS2		LODDS3		LODDS4		χ^2^	*p*^a^
	No	5-YSR	No	5-YSR	No	5-YSR	No	5-YSR	No	5-YSR		
pN stage												
N0	2524	83.1%	1331	71.3%	216	61.8%	27	38.9%			131.606	< 0.001
N1	42	70.8%	981	68.2%	956	49.7%	429	31.5%	87	18.9%	179.709	< 0.001
N2			143	68.2%	835	52.2%	772	33.3%	326	18.6%	171.909	< 0.001
N3			8	75.0%	204	47.3%	871	29.8%	1232	14.6%	152.506	< 0.001
χ^*2*^		3.416		2.981		11.473		2.625		3.991		
* p*^b^		0.065		0.395		0.009		0.453		0.136		
rN stage												
rN0	2497	82.9%	1328	71.2%	214	61.4%	25	38.4%			125.64	< 0.001
rN1	39	74.7%	907	68.7%	496	49.1%	18	33.0%			60.39	< 0.001
rN2			231	67.0%	1471	51.1%	966	32.5%	30	15.4%	138.074	< 0.001
rN3							1070	30.6%	993	17.3%	50.051	< 0.001
rN4									612	12.8%	-	-
χ^*2*^		1.411		2.493		9.203		1.742		5.81		
* p*^c^		0.235		0.287		0.01		0.628		0.055		

*LODDS* log odds of positive lymph nodes, *NO.* number of patients, *5-YSR* 5-year survival rate

^a^Comparison of overall survival rates among different LODDS groups

^b^Comparison of overall survival rates among different pN groups

^c^Comparison of overall survival rates among different rN groups

## Discussion

Despite recent advances in the treatment of patients with GC, the OS is far from satisfactory. The accuracy of staging systems for patients with GC is important for predicting long-term survival, guiding treatment, and identifying patients for clinical trials. Because of the importance of nodal involvement in assessing prognosis and defining the management of patients with GC [2, 3], there has been intense interest in defining an optimal LN staging system. In fact, numerous different parameters have been proposed to stratify the long-term prognosis of patients with GC according to the status of LN metastasis [39, 40]. The UICC/AJCC staging systems for GC stratify the nodal staging, namely pN stage, according to the number of positive LNs. The major flaw of the pN classification is that it only considers the number of metastatic LNs, but ignores the influence of the number of examined LNs, which may lead to stage migration [13, 41–44]. To overcome the potential bias associated with the pN classification, other parameters have been proposed by analyzing both the number of examined LNs and the number of metastatic LNs. The rN is a simple method for nodal staging that is less influenced by the number of examined LNs than the pN classification. Some authors have claimed that the rN classification can be an alternative to the pN classification for patients with fewer than 15 LNs examined [45–47], and the rN classification can minimize the phenomenon of stage migration [48, 49]. However, the rN0 classification was congruent with the pN0 classification in prognostic assessments of node-negative patients with GC [12, 14], and a minimum number of LNs still need to be examined to ensure accuracy for prognostic assessment. LODDS, a novel prognostic LN-related index that considers the effects of the numbers of both positive LNs and negative LNs, was developed to improve the accuracy of prognostic assessment, particularly in patients with N0 status or with < 15 total harvested LNs [50]. The LODDS classification is also considered superior to the rN and pN classifications because it can be used to study LN involvement in patients at all classification levels [11, 51–53].

This is the first meta-analysis that focused on the crucial roles of LODDS in predicting the prognosis of patients with GC. As a result, our study is more informative than any previous study. Our meta-analysis of twelve articles including 20,312 GC patients indicated that LODDS1, LODDS2, LODDS3, and LODDS4 patients have poor OS compared with LODDS0 patients, which shows that LODDS has prognostic value in patients with GC. When pooling the HR for OS, LODDS1, LODDS2, LODDS3, and LODDS4 in GC patients was correlated with poor OS compared with LODDS0 (LODDS1 vs. LODDS0: HR = 1.62, 95% CI (1.42, 1.85), *I*^*2*^ statistic = 63.5%, P _*heterogeneity*_ = 0.003; LODDS2 vs. LODDS0: HR = 2.47, 95% CI (2.02, 3.03),* I*^*2*^ statistic = 86.2%, *P *_*heterogeneity*_ < 0.001; LODDS3 vs. LODDS0: HR = 3.15, 95% CI (2.50, 3.97), *I*^*2*^ statistic = 92.1%, *P *_*heterogeneity*_ < 0.001; LODDS4 vs. LODDS0: HR = 4.55, 95% CI (3.29, 6.29),* I*^*2*^ statistic = 96.6%, P _*heterogeneity*_ < 0.001). As the LODDS grade increases, the prognosis of patients with GC becomes correspondingly worse. However, we discovered high levels of heterogeneity in all groups. To analyze the potential sources of between-study heterogeneity, we performed subgroup analysis according to differences in the variables, including the publication year, country, patient number and whether patients received neoadjuvant therapy. After subgroup analysis, it was found that the heterogeneity was mainly from whether patients received neoadjuvant therapy. Additionally, we found that patients without neoadjuvant therapy had poorer OS than patients with neoadjuvant therapy in the same group. Therefore, the results suggest that whether patients receive neoadjuvant therapy is one of the most important factors affecting the prognostic effectiveness of LODDS in patients with GC. Although we performed a subgroup analysis according to whether patients received neoadjuvant therapy, significant heterogeneity was still found in the pooled analysis of OS in the LODDS4 vs. LODDS0 group. To explore the potential sources of heterogeneity, we also used Galbraith plots and Duval and Tweedie’s trim-and-fill method to further explore the source of heterogeneity in OS, and the results showed that the training set of the study by Jian-Hui C et al. [35] might have mainly contributed substantial heterogeneity to OS.

Additionally, we assessed differences in survival among patients in different LODDS classifications for each of the pN or rN classifications. We summarized the results from four studies [11, 12, 14, 38] that compared the predictive and prognostic abilities of the LODDS staging system with those of the rN or pN classification systems. For patients in each of the rN and pN classifications, significant differences in survival were consistently observed among patients in different LODDS classifications. Meanwhile, for patients in each LODDS classification, prognosis was highly similar between patients with different pN or rN classifications (see Table 4). Thus, we considered that the superiority of the LODDS classification to the rN and pN classifications was mainly because of its potential to discriminate patients with the same ratio of node metastasis but different survival.

However, several limitations of the current meta-analysis should be emphasized. First, as several studies did not report HRs, HRs in this meta-analysis were estimated based on the method described by Tierney et al. [28]. Second, due to the lack of relevant information on the anatomic nodal group locations, we did not analyze the effect of the anatomic nodal group locations on the prognostic effectiveness of LODDS in patients with GC. Third, the optimal cutoff value of LODDS is not concluded. One critical problem is lack of consensus on the optimal cutoff value of LODDS when using it in the clinic and in experiments. Thus, LODDS is not yet used clinically on a large scale. Fourth, we were unable to apply the AUC, C-index, or AIC values to determine which of the LODDS, rN or pN classifications is superior because we did not have access to detailed patient information. Despite these limitations, this is the first meta-analysis focusing on the crucial roles of LODDS in predicting the prognosis of patients with GC. The results suggest that LODDS can predict the survival of GC patients. Moreover, it may be a novel prognostic predictor and a more accurate and sensitive stratification tool for use in clinical studies.

## Conclusion

Our systematic review demonstrated that LODDS is correlated with the prognosis of GC patients and more accurately predicts the survival of GC patients than previous methods. As the LODDS grade increases, the prognosis of patients with GC becomes correspondingly worse. Additionally, we found that patients without neoadjuvant therapy had poorer OS than patients with neoadjuvant therapy in the same LODDS group. Therefore, the results suggest that whether patients receive neoadjuvant therapy is one of the strongest factors affecting the prognostic effectiveness of LODDS. Moreover, the LODDS classification is superior to the pN and rN classifications for prognostic assessment. Incorporating LODDS into the staging system of GC will enable clinicians to predict the prognosis of patients more accurately. Further high-quality, large-scale, international, well-designed multicenter prospective studies are needed to obtain the optimal cutoff point of LODDS and to find a simple and repeatable way to calculate LODDS values to facilitate the utilization of LODDS in the clinic.

## Supplementary Information

**Additional file 1: Figure S1.** Assessment of publication bias using Funnel plot analysis. a-b Funnel plot analysis of studies on OS ((a) LODDS1 vs. LODDS0, (b) LODDS2 vs. LODDS0, (c) LODDS1 vs. LODDS0, (d) LODDS2 vs. LODDS0); e–h Funnel plot analysis of studies which included the patients without neoadjuvant therapy on OS ((e) LODDS1 vs. LODDS0, (f) LODDS2 vs. LODDS0), (g) LODDS1 vs. LODDS0, (h) LODDS2 vs. LODDS0). Publication bias was not found in the meta-analyses of OS. All of the funnel plots of the included articles showed a symmetrical distribution. Thus, no significant publication bias was found in the meta-analyses of OS. Abbreviations: HR, hazard ratio; OS, overall survival; LODDS, log odds of positive lymph nodes.

## Data Availability

All data generated or analyzed during this study are included in this published article [and its supplementary information files].

## Abbreviations

GC: Gastric cancer
LODDS: Log odds of positive lymph nodes
CI: Confidence interval
OS: Overall survival
UICC: Union for International Cancer Control
AJCC: American Joint Committee on Cancer
LN: Lymph node
LNR: Lymph node ratio
TNM: Tumor–node–metastasis
NOS: Newcastle–Ottawa scale
REM: Random-effects model
HR: Hazard ratio
HNC: Head and neck cancer
pN: Pathological N
rN: Ratio-based lymph node
