# Supplementary material for: Log odds of positive lymph nodes as a novel prognostic predictor for gastric cancer: a systematic review and meta-analysis
Source: BMC Cancer. 2023 Jun 8;23:523. doi: 10.1186/s12885-023-10805-6 (PMC10251690; doi:10.1186/s12885-023-10805-6)
Supplement: Supplementary file 1 — Additional file 1: Figure S1. Assessment of publication bias using Funnel plot analysis. a-b Funnel plot analysis of studies on OS ((a) LODDS1 vs. LODDS0, (b) LODDS2 vs. LODDS0, (c) LODDS1 vs. LODDS0, (d) LODDS2 vs. LODDS0); e–h Funnel plot analysis of studies which included the patients without neoadjuvant therapy on OS ((e) LODDS1 vs. LODDS0, (f) LODDS2 vs. LODDS0), (g) LODDS1 vs. LODDS0, (h) LODDS2 vs. LODDS0). Publication bias was not found in the meta-analyses of OS. All of the funnel plots of the included articles showed a symmetrical distribution. Thus, no significant publication bias was found in the meta-analyses of OS. Abbreviations: HR, hazard ratio; OS, overall survival; LODDS, log odds of positive lymph nodes. [file 12885_2023_10805_MOESM1_ESM.pdf]

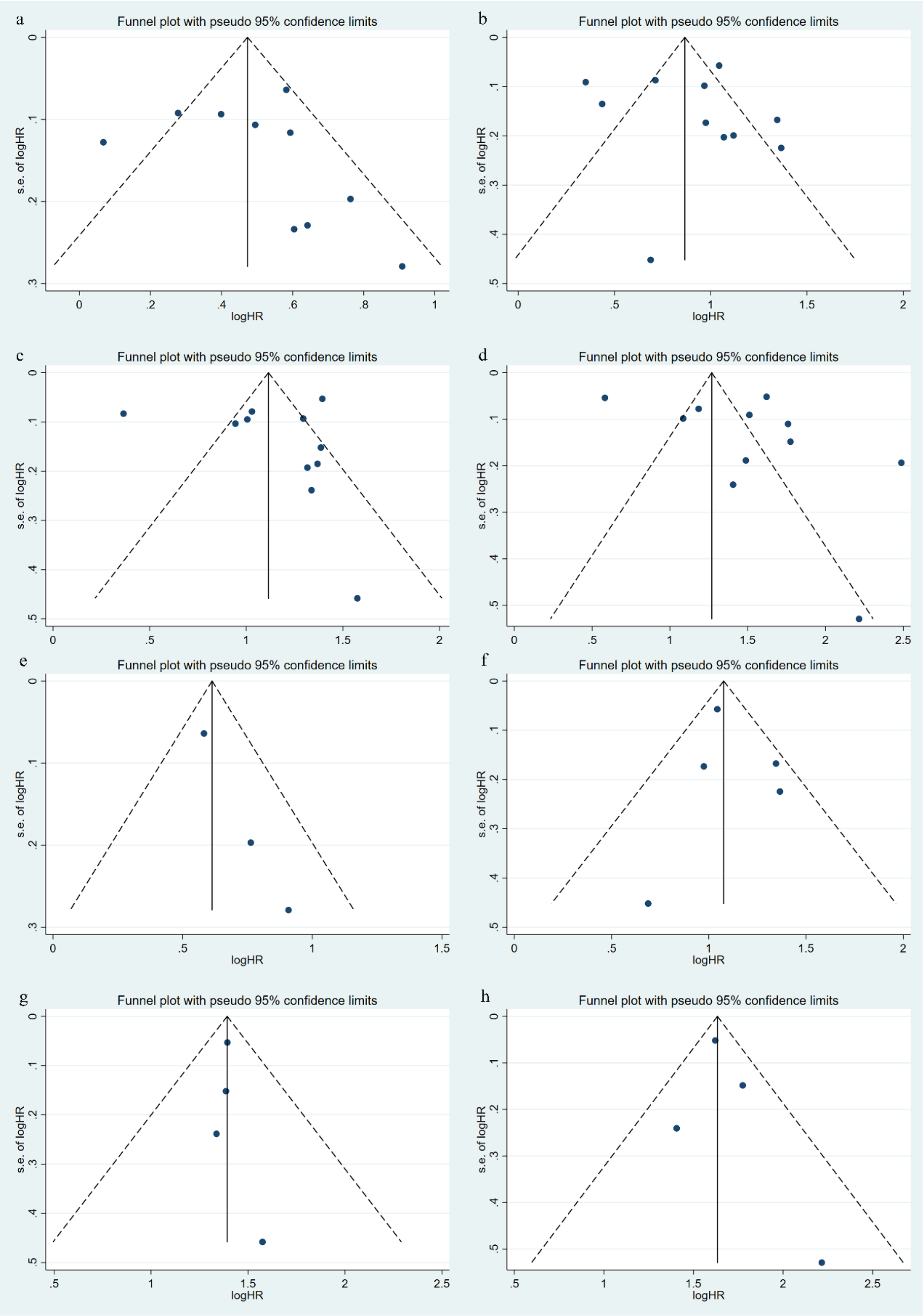


**Figure S1 Assessment of publication bias using Funnel plot analysis.** a-b Funnel plot analysis of studies on OS ((a) LODDS1 vs. LODDS0, (b) LODDS2 vs. LODDS0, (c) LODDS3 vs. LODDS0, (d) LODDS4 vs. LODDS0); e-h Funnel plot analysis of

studies which included the patients without neoadjuvant therapy on OS ((e) LODDS1 vs. LODDS0, (f) LODDS2 vs. LODDS0), (g) LODDS3 vs. LODDS0, (h) LODDS4 vs. LODDS0). Publication bias was not found in the meta-analyses of OS. All of the funnel plots of the included articles showed a symmetrical distribution. Thus, no significant publication bias was found in the meta-analyses of OS. Abbreviations: HR, hazard ratio; OS, overall survival; LODDS, log odds of positive lymph nodes.
